# The complete chloroplast genome sequence of *Epipremnum aureum* and its comparative analysis among eight Araceae species

**DOI:** 10.1371/journal.pone.0192956

**Published:** 2018-03-12

**Authors:** Na Tian, Limin Han, Chen Chen, Zhezhi Wang

**Affiliations:** 1 National Engineering Laboratory for Resource Development of Endangered Crude Drugs in Northwest of China, Key Laboratory of Ministry of Education for Medicinal Resources and Natural Pharmaceutical Chemistry, College of Life Sciences, Shaanxi Normal University, Xi’an, Shaanxi, P.R. China; 2 Department of Bioscience and Biotechnology, Shaanxi Xueqian Normal University, Xi’an, Shaanxi, P.R. China; 3 Institute of Botany of Shaanxi Province, Xi’an Botanical Garden of Shaanxi Province, Xi’an, Shaanxi, P.R. China; Austrian Federal Research Centre for Forests BFW, AUSTRIA

## Abstract

*Epipremnum aureum* is an important foliage plant in the Araceae family. In this study, we have sequenced the complete chloroplast genome of *E*. *aureum* by using Illumina Hiseq sequencing platforms. This genome is a double-stranded circular DNA sequence of 164,831 bp that contains 35.8% GC. The two inverted repeats (IRa and IRb; 26,606 bp) are spaced by a small single-copy region (22,868 bp) and a large single-copy region (88,751 bp). The chloroplast genome has 131 (113 unique) functional genes, including 86 (79 unique) protein-coding genes, 37 (30 unique) tRNA genes, and eight (four unique) rRNA genes. Tandem repeats comprise the majority of the 43 long repetitive sequences. In addition, 111 simple sequence repeats are present, with mononucleotides being the most common type and di- and tetranucleotides being infrequent events. Positive selection pressure on *rps12* in the *E*. *aureum* chloroplast has been demonstrated via synonymous and nonsynonymous substitution rates and selection pressure sites analyses. *Ycf15* and *infA* are pseudogenes in this species. We constructed a Maximum Likelihood phylogenetic tree based on the complete chloroplast genomes of 38 species from 13 families. Those results strongly indicated that *E*. *aureum* is positioned as the sister of *Colocasia esculenta* within the Araceae family. This work may provide information for further study of the molecular phylogenetic relationships within Araceae, as well as molecular markers and breeding novel varieties by chloroplast genetic-transformation of *E*. *aureum* in particular.

## Introduction

Chloroplasts are organelles in green plants and algae, where they are responsible for photosynthesis and other housekeeping functions that are essential for nitrate and sulphate assimilation, as well as the synthesis of amino acids, fatty acids, chlorophyll, and carotenoids [1, 2]. Over the long period time of evolution, the sizes of chloroplasts genomes have reduced dramatically to 120–130 genes in recent times, due to gene loss and/or the large-scale gene transfer to the nuclear genome [1, 3]. Chloroplast DNA sequences were first discovered during the process of physically mapping the *Zea mays* chloroplast [4]. Since the first complete nucleotide sequences were reported for *Marchantia polymorpha* and tobacco (*Nicotiana tabacum*) [5, 6], complete chloroplast (cp) genomes have been determined for numerous other species. The cp genomes for more than 1000 land plants are now available from the NCBI (National Center for Biotechnology Information) Organelle Genome Resources, including those of ornamental plants such as *Wisteria floribunda* and *Magnolia kwangsiensis* [7–9]. Most of these deposited genomes typically possess a quadripartite structure, i.e., a pair of inverted repeats (IRa and IRb) divided by a large single-copy (LSC) region and a small single-copy (SSC) region [3, 10]. These cp genomes are widely used in research on comparative genomics, plant system development, and phylogeny at different taxonomic orders. When combined with information from the mitochondrion and nucleus, these cp data can help resolve undetermined relationships for plants at the genus or higher taxon level [2, 11–13].

*Epipremnum aureum*, commonly known as ‘pothos’, is a large perennial vine plant that originated from Southeast Asia and the Solomon Islands in Indonesia [14]. It is the only shy-flowering species known in Araceae, a large, diverse family that contains 120 genera and 3,800 species [15]. Despite the long and arduous process of nomenclature with conventional morphological identifications since 1880, formerly known as ‘*Pothos aureus*’, ‘*Scindapsus aureus*’, ‘*Raphidophora aurea*’, or ‘*Epipremnum pinnatum’*, was named as *Epipremnum aureum* in 1964 when flower characteristics were available [15]. Its decorative marbled leaves and ease of maintenance make it very popular as an indoor plant because it is tolerant of low light levels and has beautiful evergreen foliage, air-cleansing qualities, and desirable vastu principles [14, 16, 17]. Plants of this species also possess antibacterial, anti-termite, and antioxidant properties, as well as components that can help in healing wounds and protecting humans against malaria, cancer, tuberculosis, and arthritis [18]. This species was first marketed as an ornamental in the United States in the 1920s, and was considered the second-most popular foliage plant from the 1950s to 1960s. By 2007, however, its ranking had dropped to twelfth place because only a limited number of cultivars were in commercial production [14]. Because it is recalcitrant in its flowering nature, breeding protocols that rely upon floral induction for propagation are not yet available [14, 15]. Furthermore, induced mutation as a method for addressing the shortage of new cultivars has not been successful because of low frequency and randomness [14, 15]. Genetic engineering can possibly provide a more efficient means for cultivating new cultivars through gene(s) transfer. Such efforts are based upon the cp genome, an approach that has several advantages when compared with nuclear genome transformation, e.g., high-level expression or a lack of gene silencing [14, 19, 20]. However, the limited number of cp genome sequences that have been completed represents a major factor that hinders the application of chloroplast genetic engineering to useful crops even though many plant genomes have already been sequenced or are in progress [19, 21].

The predominantly aquatic order Alismatales includes three clades: Araceae, Tofieldiaceae, and Alismatiflorae, and its members are widely distributed, highly diverse group that contains 14 families, 166 genera, and approximately 4,500 species that are important to the study of origins and early diversification of monocotyledonous plants [22]. The 118 genera and ca 3,800 species in Araceae are distributed mostly in the tropics and subtropics of the northern and southern hemispheres [18]. They are aquatic, epiphytic, climbing or terrestrial plants that have a wide range of habitats with diverse morphological characteristics [23]. Together with the advancement of molecular evolution and phylogenetics, the classification of Araceae and its order (Alismatales) based on conventional morphological studies have been modified frequently; the relationships amongst them (even alismatids) have not been clarified completely because of the continuous development and changes, such as the placement of *Calla*, *Acorus* and Lemnaceae [23–27]. Here, we assembled the cp genome of *E*. *aureum* and compared it with that of other plants within Araceae. We then constructed a phylogenetic tree to make comparisons with cp genomes published for other plant species in related families. Our objectives were to provide information for the systematic evolution studies of Araceae and Alismatales, with special interest in the positioning of *E*. *aureum* in plant systematics and evolution. We also investigated intergenic spacer and regulatory sequences to obtain information that can be used in future studies for chloroplast genetic engineering.

## Materials and methods

### DNA extraction, genome sequencing, assembly, and annotation

Fresh leaves of *Epipremnum aureum* were collected and total DNA was prepared following the instruction of Gentra Puregene Tissue Kit (QIAGEN Biotechnology, Germany), for constructing a shotgun library and sequencing on an Illumina Hiseq 2500 platform (Genesky Biotechnologies, Inc., Shanghai, China). The raw data (1.41 G) were filtered and trimmed by CLC Genomics Workbench version 7.5 (CLC Bio, Aarhus, Denmark). In all, 793,730 reads were assembled with MITobim version 1.7 [28] according to the complete cp genome sequence of *Colocasia esculenta* (KC016753). For filling the gaps, the high-quality flanking sequences (~600 bp of each end) of gaps were selected as a starting seed for the direct reconstructing process, respectively. The gaps could be fixed by aligning the new consensus sequences with the initial assembled cp genome sequence. At last, the quality checks were implemented by mapping the trimmed reads to the assembled cp genome sequence. The assembled sequence was annotated by GENEIOUS R8 (Biomatters Ltd., Auckland, New Zealand), with the complete cp genome sequence of *Colocasia esculenta* (KC016753) serving as the reference [29]. Statistical analyses of synonymous codon usage for all protein-coding genes and gene sequence alignments were also performed with GENEIOUS R8.

### Comparative genome analysis

The synonymous (dS) and nonsynonymous (dN) substitutions of chloroplast genomes in members of Araceae were evaluated by BioEdit [30, 31] and DnaSPs software [31, 32]. The selection pressure sites analysis of *rps12* in Araceae species (*Colocasia esculenta*, *Epipremnum aureum*. *Dieffenbachia seguine*, *Lemna minor*, *Pinellia ternata*, *Spirodela polyrhiza*) was performed by Datamonkey (http://www.datamonkey.org/). A comparative analysis was performed with complete cp genome sequences from *Colocasia esculenta*, *Epipremnum aureum*. *Dieffenbachia seguine*, *Lemna minor*, *Pinellia ternata*, *Spirodela polyrhiza*, *Wolffia australiana*, and *Wolffiella lingulata*. These eight genomes were compared by the mVISTA tool and the Shuffle-LAGAN alignment algorithm [33], using the cp genome of *Camellia crapnelliana* as our reference.

### Identification of repeats

Repeats in the *E*. *aureum* cp genome were detected as described by Chen et al. [10]. Tandem repeats were identified by Tandem Repeats Finder version 4.07b [34] with default settings. Forward repeats and palindromic repeats were determined by REPuter [35], with the minimum size set at 30 bp and a Hamming distance of 3. Simple sequence repeats (SSRs) were examined by Perlscript MISA2 (http://pgrc.ipk-gatersleben.de/misa/misa.html) to detect mono-, di-, tri-, tetra-, penta-, and hexa nucleotide motifs, with the thresholds of repeat units set at 8, 4, 4, 3, 3, and 3, respectively. The repeats obtained via these different software programs were filtering by hand to reject unnecessary details.

### Phylogenetic analysis

The plastid genomic sequences of *E*. *aureum* and 37 other species were collected from NCBI (S1 Table). The coding sequences (CDS) of 53 common protein-coding genes (S1 File) from the 38 species were aligned with the MAFFT method based on codons by GENEIOUS R8. The final alignment was concatenated by the 53 CDS alignments (S2 File). The best nucleotide substitution model (GTR+G+I) was tested and a Maximum Likelihood (ML) tree (1000 bootstrap replicates) was constructed with MEGA 6.0 software [36].

## Results and discussion

### Genome assembly and analysis of genetic features

**Fig 1:**
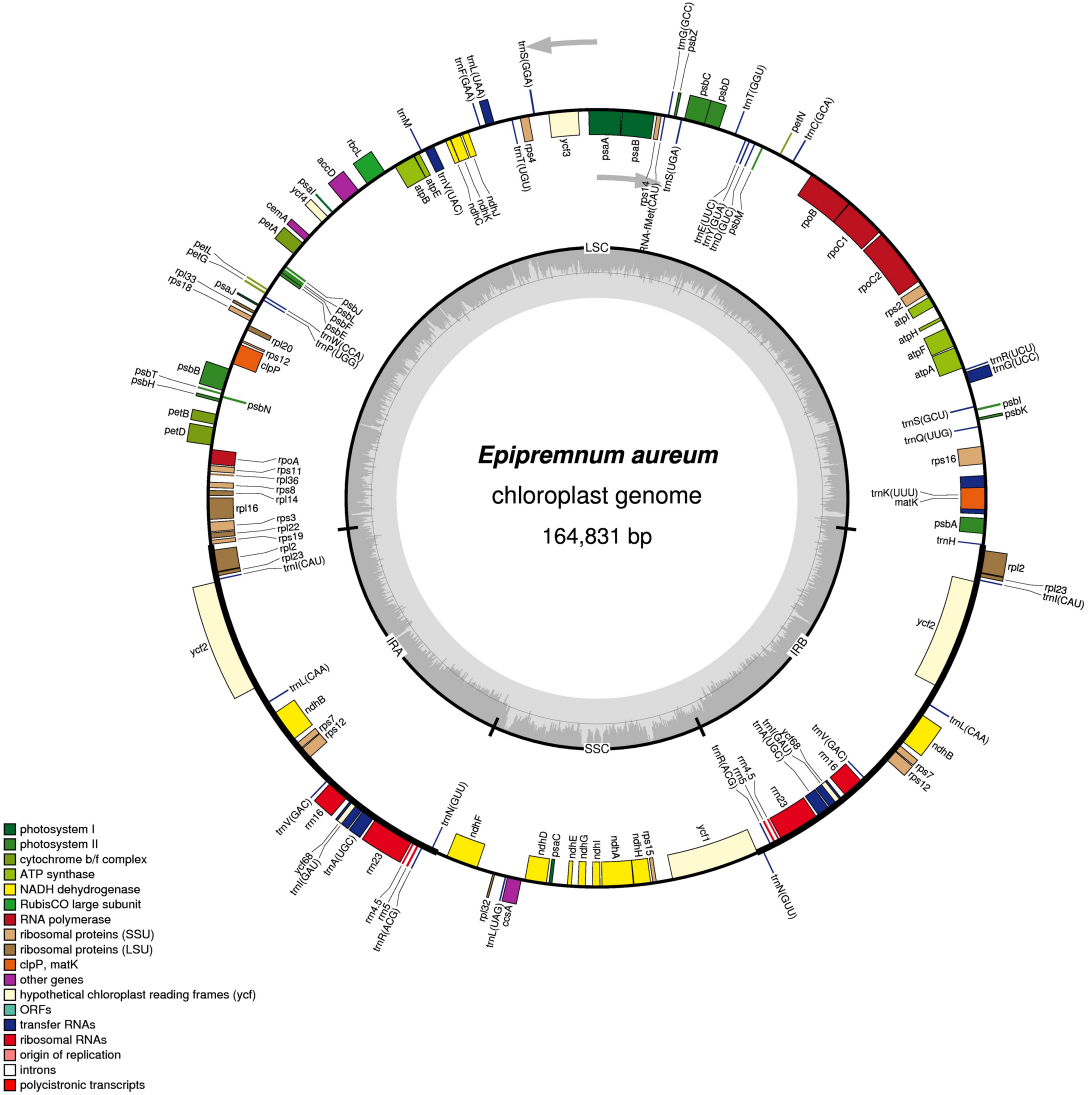
Gene distribution map for cp genome of *Epipremnum aureum*. Genes in inner and outer rings are transcribed in clockwise and counter-clockwise directions, respectively. Functional gene groups are indicated by different colors. For inner circles, dark grey and light grey represent GC and AT content, respectively.

In total, 19,318,116 reads, averaging 125 bp long, were obtained on the Illumina HiSeq2500 platform. From them, 793,730 reads were used to assemble the complete chloroplast genome of *Epipremnum aureum*. Its double-stranded circular DNA sequence consists of 164,831 bp (Fig 1), and has been deposited in GenBank with Accession Number KR872391. The 35.8% GC content is similar to that reported for other plants, including 38.3% for *Wisteria floribunda*, *Magnolia kwangsiensis*, and *Andrographis paniculata* [7, 8, 37]. The *E*. *aureum* genome presents a common quadripartite structure of similar size to the majority of angiosperm species, and consists of two IR regions (IRa and IRb; each 26,606 bp) that are separated by the LSC region (88,751 bp) and the SSC region (22,868 bp) [7, 8, 37, 38]. Its sequence contains protein-coding regions (49.1%), rRNA (5.6%), tRNA (1.7%), and non-coding sequences (43.6%, including introns and intergenic spacers, or IGS). The AT and GC contents are 64.2% and 35.8%, respectively (Table 1).

**Table 1:** Comparison of chloroplast sequences for *E*. *aureum* and other species within Araceae family.

Species	Accession Number	Size (bp)	LSC		SSC		IR		Total	
			Length (bp)	GC (%)	Length (bp)	GC (%)	Length (bp)	GC (%)	AT (%)	GC (%)
*Colocasia esculenta*	JN105689	162,424	89,670	34.31	22,208	28.95	25,273	42.44	63.84	36.16
*Dieffenbachia seguine*	KR262889	163,699	90,780	34.65	22,440	29.31	25,235	42.66	63.61	36.39
*Epipremnum aureum*	KR872391	164,831	88,751	33.81	22,868	29.18	26,606	41.89	64.22	35.78
*Pinellia ternata*	KR270823	164,013	89,783	34.60	22,980	31.66	25,625	42.53	63.34	36.66
*Lemna minor*	DQ400350	165,955	89,907	33.51	13,603	30.34	31,223	40.06	64.28	35.72
*Spirodela polyrhiza*	JN160603	168,788	91,222	33.48	14,056	30.18	31,755	40.08	64.31	35.69
*Wolffia australiana*	JN160605	168,704	91,454	33.80	13,392	30.76	31,930	39.89	64.14	35.86
*Wolffiella lingulata*	JN160604	169,337	92,015	33.72	13,956	30.79	31,683	40.03	64.16	35.84

This genome contains 131 (113 unique) functional genes, including 86 protein-coding genes (79 unique plus *ycf1*, *ycf2*, *ycf3*, *ycf4*, and *ycf68* as hypotheticals), 37 (30 unique) tRNA genes, and eight (four unique) rRNA genes. Furthermore, 17 genes are replicated in both IR regions, i.e., six protein-coding genes and 11 RNA genes (four rRNA and seven tRNA) (Table 2). The LSC region has 60 protein-coding and 22 tRNA genes while the SSC region has 12 protein-coding and one tRNA gene (Fig 1). Introns occur in 19 genes, with 17 of them–*atpF*, *pet*(*B*, *D*), *ndh*(*A*, *B*), *rpl*(*2*, *16*), *rps*(*12*, *16*), *rpoC1*, *trn*(*A-UGC*, *G-GCC*, *I-GAU*, *K-UUU*, *L-UAA*, *V-UAC*), and *ycf68—*having one intron each while two genes–*clpP* and *ycf3*—have two introns each (Table 3). Another gene, *rps12*, is unusual because it has three exons, with the 5’ exon occurring in the LSC region and two 3’ exons being repeated in the IRa and IRb regions [39]. That gene is considered to be trans-spliced in the cp genomes [3, 4, 13]. Similar to *Metasequoia glyptostroboides* [10], the largest intron in *E*. *aureum* (2,554 bp) is found in *trnK-UUU*. These features of the *E*. *aureum* cp genome are quite universal in land plants [3].

**Table 2:** Gene groups with different functions in the chloroplast genome of *E*. *aureum*.

Function	Gene group	Gene name
Photosynthesis pathways	Photosystem I	*psa*(*A*, *B*, *C*, *I*, *J*)
	Photosystem I assembly	*ycf*(3**, *4*)
	Photosystem II	*psb*(*A-F*, *H-N*, *T*, *Z*)
	F-type ATP synthase	*atp*(*A*, *B*, *E*, F*, *H*, *I*)
	NAD(P)H-dehydrogenase Complex	*ndh*(A*, B*^#^, *C-K*)
	Component of cytochrome b6/f complex	*pet*(*A*, B*, D*, *G*, *L*, *N*)
	Inner envelope membrane	*cemA*
	Cytochrome c biogenesis protein	*ccsA*
	Large subunit of Rubisco	*rbcL*
Structural RNAs	Transfer RNAs	*trn*(A-UGC*^#^, *C-GCA*, *D-GUC*, *F-GAA*, *fM-CAU*, *G-GCC*, G-UCC*, *H*, *I-CAU*^#^, I-GAU*^#^, K-UUU*, *L-CAA*^#^, L-UAA*, *L-UAG*, *M*, *N-GUU*^#^, *P-UGG*, *Q-UUG*, *R-ACG*^#^, *R-UCU*, *S-GCU*, *S-GGA*, *S-UGA*, *T-GGU*, *T-UGU*, *V-GAC*^#^, V-UAC*, *W-CCA*, *Y-GUA*, *E-UUC*)
	Ribosomal RNAs	*rrn*(*4*.*5*^#^, *5*^#^, *16*^#^, *23*^#^)
Genetic apparatus	Large subunit of ribosomal protein	*rpl*(2*c, *14*, 16*, *20*, *22*, *23*^#^, *32*, *33*, *36*)
	Small subunit of ribosomal protein	*rps*(*2*, *3*, *4*, *7*^#^, *8*, *11*, 12*^&^, *14*, *15*, 16*, *18*, *19*^#^)
	Subunits of RNA polymerase	*rpo*(*A*, *B*, C1*, *C2*)
Post-transcriptional modification	Maturase	*matK*
Protein-modifying	ATP-dependent Clp protease proteolytic subunit	clpP**
Biosynthesis of fatty acids	Acetyl-CoA carboxylase	*accD*
Unknown Proteins		*ycf*(*1*, *2*^#^, 68*^#^)

*, 1 intron in gene;

**, 2 introns in gene;

^#^, gene repeated in IR region;

^&^, gene has 2 separate transcription units.

**Table 3:** Split genes and size of introns in the chloroplast genome of *E*. *aureum*.

Gene	Location	Exon1 (bp)	Intron1 (bp)	Exon2 (bp)	Intron2 (bp)	Exon3 (bp)
rps12*	LSC-IRs	41	–	152		
*rps16*	LSC	40	994	197		
*rpoC1*		1629	737	459		
*atpF*		145	823	401		
*ycf3*		153	795	230	741	124
*clpP*		340	480	46	501	89
*petB*		6	56	642		
*petD*		8	733	475		
*rpl16*		399	1085	9		
*trnL-UAA*		37	505	50		
*trnV-UAC*		36	597	39		
*trnK-UUU*		37	2552	42		
*rpl2*	IR	434	692	391		
*ndhB*		777	695	759		
*trnI-GAU*		42	949	35		
*trnA-UGC*		38	799	35		
*trnG-UCC*		24	715	48		
*ycf68*		42	31	303		
*ndhA*	SSC	553	1077	539		

*rps12 is a trans-spliced gene containing three exons: the first exon is in the LSC region while the second and third exons are located in both IR regions [39].

### Comparisons of chloroplast genomes among eight species in Araceae

**Fig 2:**
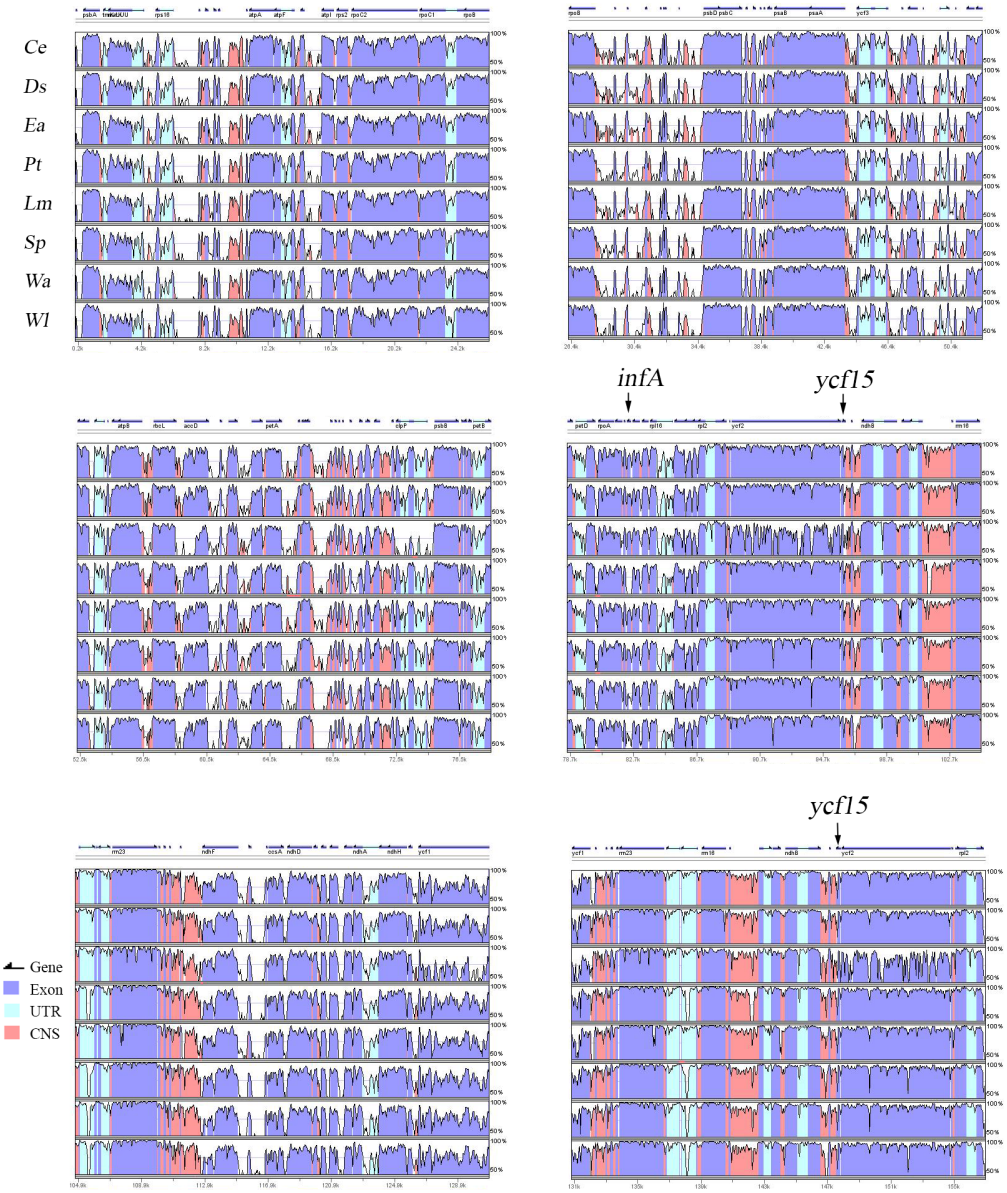
Alignment map of 8 cp genomes from species within Araceae. Vertical axis, identities of sequences from 50% to 100%. Horizontal axis, coordinates within cp genome. Arrows indicate genes and transcriptional directions. Exons, conserved non-coding sequences (CNS), and untranslated regions (UTRs) are shown in different colors. Species abbreviations: *Ce*, *Colocasia esculenta*; *Ds*, *Dieffenbachia seguine*; *Ea*, *Epipremnum aureum*; *Pt*, *Pinellia ternata*; *Lm*, *Lemna minor*; *Sp*, *Spirodela polyrhiza*; *Wa*, *Wolffia australiana*; and *Wl*, *Wolffiella lingulata*.

We compared the cp genome sequences of *E*. *aureum* and other species in the Araceae family and found similar GC contents that ranged from 35.66 to 36.39% (Table 1), which is within the range of 31.0 to 38.0% for typical monocots [31]. Within the Araceae family, the cp genomes are larger for four floating plants (*L*. *minor*, *S*. *polyrhiza*, *W*. *australiana*, and *W*. *lingulata*) than for four terrestrial plants (*C*. *esculenta*, *E*. *aureum*, *P*. *ternata*, and *D*. *seguine*) (Table 1). We used mVISTA to study these variations and confirmed that the overall gene content and order in the cp genome of *E*. *aureum* is similar to those in other Araceae members (Fig 2), without inversions or translocations. However, the greatest variations were observed in four genes (*clpP*, *accD*, *ycf1*, and *ycf2*) in the coding regions for *E*. *aureum* and *Dianthus superbus* [40]. In addition, one copy each of *ycf1* and *rps15* is located in the IRs for our four floating plants whereas those genes are within the SSC regions for the four terrestrial plants. As shown in Fig 2, a feature common to the genomes of land plants is that genes are much more highly conserved in their coding regions than in their non-coding regions [3, 40].

#### *ycf1* and *ycf2*

During the evolution of plastids, genome sizes and individual plant genes have changed, with many functional genes being lost or transferred to nuclear or mitochondrial genomes [3]. For example, the remarkable reduction in genome size and gene contents in *Epifagus* (a non-photosynthetic parasitic species) is due to the uselessness of most photosynthesis and expression-related genes [3, 41]. The possible reason of genome reduction is that the tactic could save resources for their DNA replication quickly under adverse environmental conditions, e.g. genes lost from Pinaceae, Gnetophytes, and intron and spacer regions reduction in *Gnetum* and *Welwitschia* [3, 41]. Among the 85 *ycf* (hypothetical open reading frame) genes found in plant cp genomes, functions are still unclear for most of them, and only 18 genes have been characterized as being involved in photosynthesis or other essential processes [42]. In contrast to genes that have been lost, both *ycf1* and *ycf2* have possibly arisen from an ancestor common to land plants and some green algae [3, 43]. In decades, researchers have paid much attention to *ycf1* and *ycf2*, the two largest open reading frames in chloroplast genomes of higher plants, which are thought to participate in various cellular activities related to ATPase, chaperones, cell division, structural remodeling, transport of proteins across membranes, or proteolysis [3, 44–47]. In *Arabidopsis*, *ycf1* is functionally classified as coding TIC214 (a translocon at the chloroplast inner envelope membrane), which is considered essential for plant survival because most exotic proteins are imported into chloroplasts via Tic214/Tic20 [48]. Moreover, the loss of *ycf1* genes from monocotyledonous species (e.g., Poales, Acorales, and some parasitic plants) and dicotyledons such as *Vaccinium* sp. indicates that YCF1 does not act as a TIC in other plants as it does in *Arabidopsis* [3, 48–50], or that is possibly transferred to the nucleus in these species. In contrast, Nakai et al suggest YCF1 is a green TIC and largely conserved among the Chlorophyta and land plants [51]. Hence, whether *ycf1* is a bona fide green TIC is still controversial.

#### *InfA*, *ycf15*, and *ycf68*

**Fig 3:**
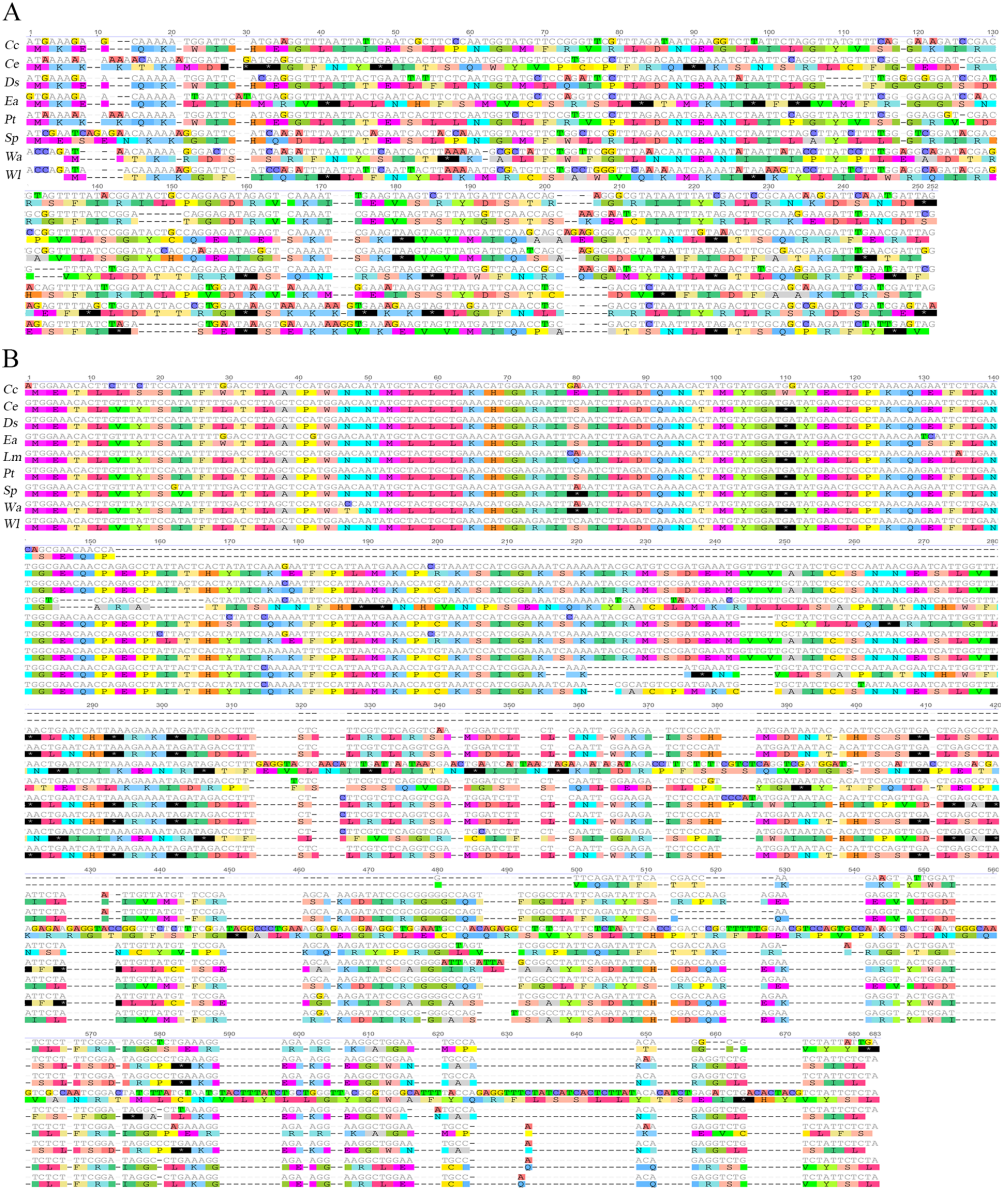
Nucleotide alignments of *InfA* (A) and *ycf15* (B) in *Colocasia esculenta* (*Ce*), *Dieffenbachia seguine* (*Ds*), *Epipremnum aureum* (*Ea*), *Pinellia ternata* (*Pt*), *Lemna minor* (*Lm*), *Spirodela polyrhiza* (*Sp*), *Wolffia australiana* (*Wa*), and *Wolffiella lingulata* (*Wl*), with *Camellia crapnelliana* (*Cc*) as reference.

Our global comparison via mVISTA also revealed homologous fragments of *infA* and *ycf15* in the cp genomes from the majority of Araceae members (Fig 2). However, except for the loss of *infA* in *L*. *minor*, both appear to be pseudogenes based on the presence of stop codons in those sequences (Fig 3A and 3B). Therefore, the cp genome of *E*. *aureum* has two pseudogenes: one *infA* and two *ycf15* (in IR regions). Our results are consistent with those reported for *infA* and *ycf15* in *C*. *esculenta* [29]. *infA* is a functional gene in more than 300 angiosperm chloroplasts, which encodes translation initiation factor 1 [43]. During evolution, it was lost entirely or persists as a pseudogene in most angiosperm, or it was transferred to nucleus in some species [43, 52]. The *ycf15*, with unknown function, is usually located downstream of *ycf2*, with an intact or interrupted form in a small group of land plants [3, 53]. The role of *ycf15* in *Spinacia oleracea* may be similar to that of *sprA* in tobacco, acting as a regulatory sequence or specifying a structural RNA that is non-essential for normal growth, but which involves complex post-transcriptional splicing and does not function as a protein-coding gene [3, 53–55].

Similarly, the function of *ycf68* is also unclear. This gene occurs intact in the IR regions of our four tested land plants but is a pseudogene in the four floating plants examined here. Therefore, expression of *ycf68* might be related to plant adaptations to terrestrial circumstances. Other research results have suggested that it is a protein-coding gene in plants such as *Pinus thunbergii*, *P*. *koraiensis*, Nymphaeales, and grass species; that it participates as an excising intro or regulatory gene in non-protein processes for *Aneura mirabilis* and several angiosperms; or else exists as a pseudogene in most other species [3, 53]. Both *Ycf15* and *ycf68* are conserved when occurring in the slowly evolving IR region, but not because of any functional requirement [46, 53].

#### Codon usage

All 79 protein-coding genes in the *E*. *aureum* cp genome are encoded by 27,023 codons and 86 stop codons (36, TAA; 28, TAG; and 22, TGA; Table 4). The dominant start codon is ATG, substituting for CTG in *accD* and *rps19*, ACG in *ndhD* and *rpl2*, and ATA in *rpl22*. In most angiosperm plasmids, the start codons of *rpl2* and *rps19* are common RNA editing sites where ACG changes to AUG [2, 31]. The ACG editing site in *ndhD* transcripts might have been lost through the slow rate of evolution or a C mutation back to T in the *ndhD* start codon. Thus, this loss of ACG as the start codon in *ndhD* might seriously influence the accumulation of NDH complex in the leaves [2].

**Table 4:** Relative synonymous codon usage (RSCU) for protein-coding genes in *Epipremnum aureum*.

Codon	AA	Freq	RSCU	Codon	AA	Freq	RSCU
GCA	A (Ala)	426	1.192	AAT	N (Asn)	984	1.545
GCC	A (Ala)	227	0.635	CCA	P (Pro)	337	1.182
GCG	A (Ala)	154	0.431	CCC	P (Pro)	217	0.761
GCT	A (Ala)	622	1.741	CCG	P (Pro)	142	0.498
TGC	C (Cys)	83	0.494	CCT	P (Pro)	444	1.558
TGT	C (Cys)	253	1.506	CAA	Q (Gln)	700	1.480
GAC	D (Asp)	225	0.416	CAG	Q (Gln)	246	0.520
GAT	D (Asp)	858	1.584	AGA	R (Arg)	565	1.979
GAA	E (Glu)	1,008	1.427	AGG	R (Arg)	185	0.648
GAG	E (Glu)	405	0.573	CGA	R (Arg)	337	1.180
TTC	F (Phe)	546	0.688	CGC	R (Arg)	119	0.417
TTT	F (Phe)	1,042	1.312	CGG	R (Arg)	129	0.452
GGA	G (Gly)	700	1.594	CGT	R (Arg)	378	1.324
GGC	G (Gly)	176	0.401	AGC	S (Ser)	109	0.317
GGG	G (Gly)	302	0.688	AGT	S (Ser)	442	1.284
GGT	G (Gly)	579	1.318	TCA	S (Ser)	429	1.246
CAC	H (His)	165	0.517	TCC	S (Ser)	347	1.008
CAT	H (His)	473	1.483	TCG	S (Ser)	172	0.500
ATA	I (Ile)	772	0.996	TCT	S (Ser)	566	1.645
ATC	I (Ile)	450	0.580	ACA	T (Thr)	405	1.197
ATT	I (Ile)	1,104	1.424	ACC	T (Thr)	238	0.704
AAA	K (Lys)	1,117	1.464	ACG	T (Thr)	178	0.526
AAG	K (Lys)	409	0.536	ACT	T (Thr)	532	1.575
CTA	L (Leu)	366	0.784	GTA	V (Val)	532	1.470
CTC	L (Leu)	202	0.433	GTC	V (Val)	204	0.564
CTG	L (Leu)	165	0.354	GTG	V (Val)	195	0.539
CTT	L (Leu)	595	1.275	GTT	V (Val)	517	1.428
TTA	L (Leu)	895	1.918	TGG	W (Trp)	444	1.000
TTG	L (Leu)	577	1.236	TAC	Y (Tyr)	221	0.419
ATA	M (Met)	1	0.005	TAT	Y (Tyr)	835	1.581
ATG	M (Met)	599	2.985	TAA	TAA (*)	36	1.256
CTG	M (Met)	2	0.010	TAG	TAG (*)	28	0.977
AAC	N (Asn)	290	0.455	TGA	TGA (*)	22	0.767

*, stop codon

**Fig 4:**
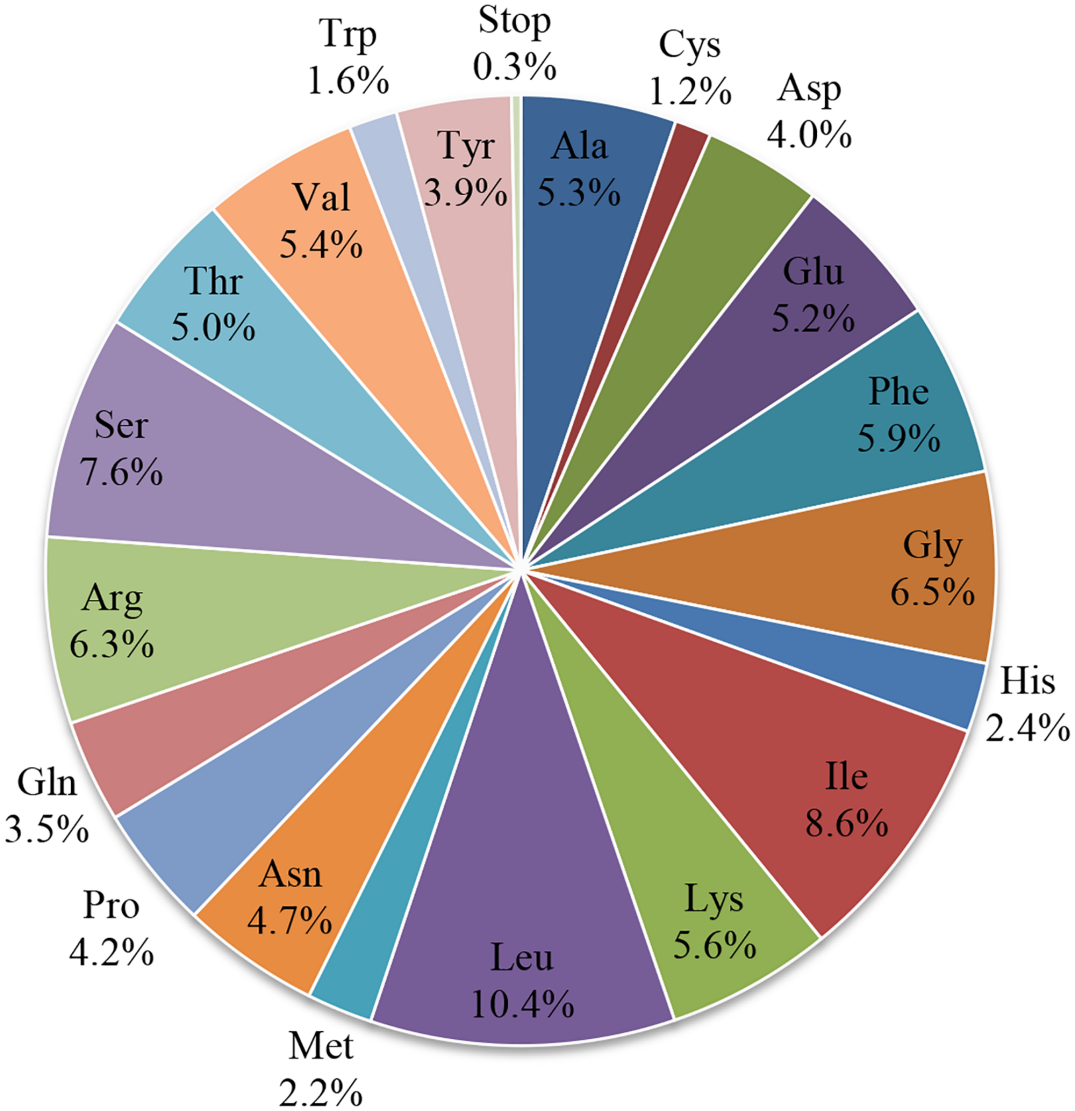
Composition of amino acid codons for protein-coding genes in *Epipremnum aureum* chloroplast genome.

Amino acids leucine (10.4%) and cysteine (1.2%) are found in the highest and lowest proportions, respectively (Fig 4), while the codons of AAA (1117) for lysine and UGC (83) for cysteine occur in the highest and lowest proportions, respectively. These features of leucine (10.4%) and UGC (83) for cysteine have also been reported for the cp genomes of *Colchicum autumnale*, *Gloriosa superba*, and *Metasequoia glyptostroboides* [10, 31]. However, except for *E*. *aureum*, AAA (1117) is the most widely used codon in these species [10]. The codon usage of *E*. *aureum* is rather similar to that of *Nelumbo nucifera* and *Colchicum autumnale* [2, 31]. Our relative synonymous codon usage (RSCU) results (Table 4) demonstrate that codons in the *E*. *aureum* cp genome at the third position have a higher nucleotide frequency for A or T than for G or C. Furthermore, the calculated RSCUs for alanine show that the values for GCC (0.635) and GCG (0.431) are dwarfed by those of GCA (1.193) and GCT (1.741). In the cp genomes of numerous land plants, it is common for the preference of AT at the third codon position to be positively correlated with the contents of A and T [10].

### Simple sequence repeats and long repetitive sequences

**Fig 5:**
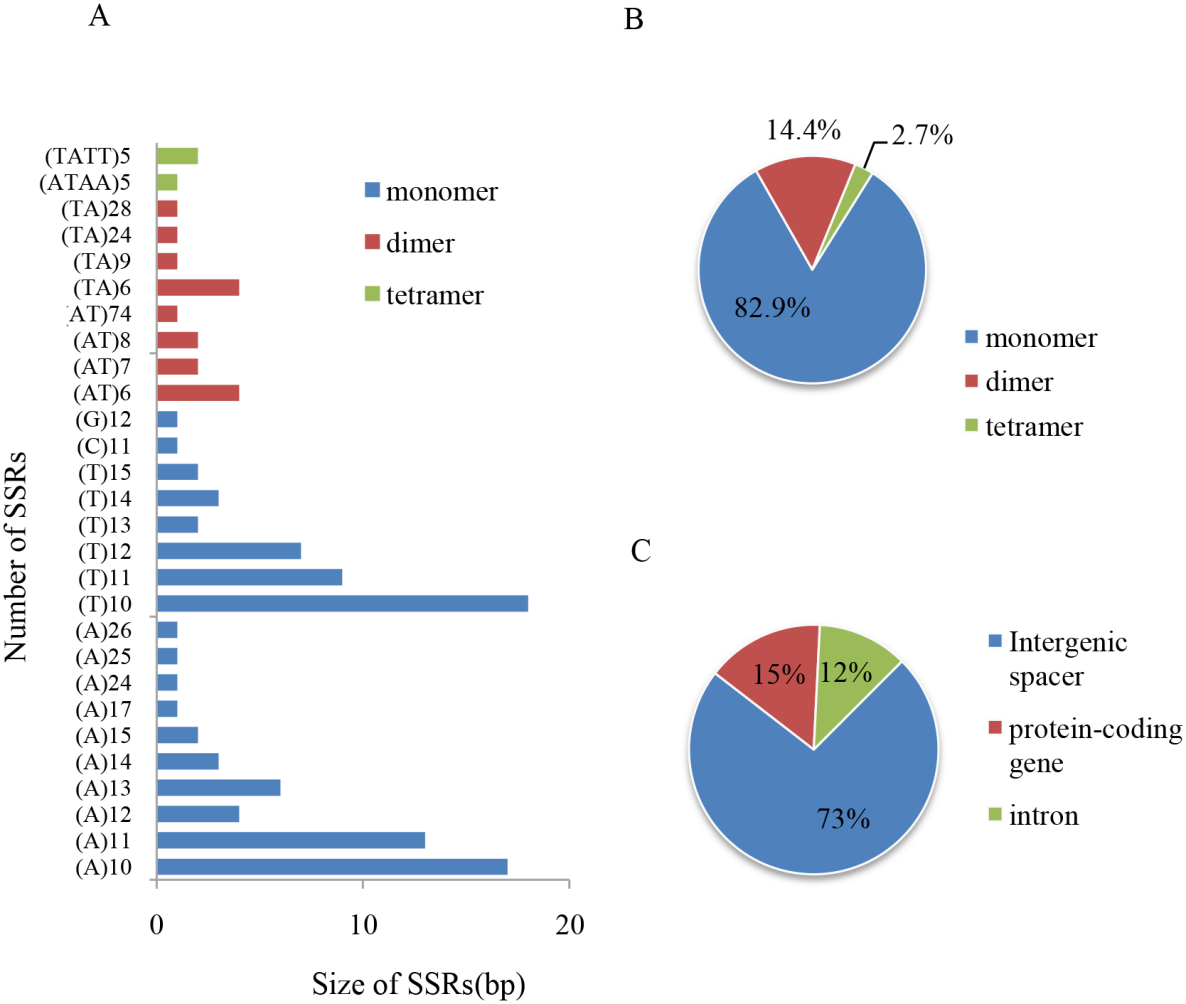
Statistical analysis of SSRs in *Epipremnum aureum* chloroplast genome. (A) Sorted by type of SSR. (B) Frequency by type. (C) Sorted by region of genome.

Simple sequence repeats, also known as microsatellites or short tandem repeats, are 1- to 6-bp repeating sequences that are widely distributed over a genome. Because of their co-dominance and high degree of polymorphism, SSRs are useful genetic markers for research involving gene flow, population genetics, molecular breeding, and gene mapping [10]. The cp genome of *E*. *aureum* (S2 Table) includes 111 SSRs longer than 10 bp that are repeated 5 to 74 times (Fig 5A). Mononucleotides, dinucleotides, and tetranucleotides account for 82.9%, 14.4%, and 2.7%, respectively, of those SSRs (Fig 5A and 5B). The majority (98.9%) of the mononucleotide repeats is AT-rich. Likewise, all of the dinucleotides and tetranucleotide are AT-rich, being composed of AT/TA and ATAA/TATT repeats, respectively (Fig 5A). These results demonstrate that chloroplast SSRs usually consist of short repeats of polyA/T [31]. The high A/T frequency in chloroplast SSRs also contributes to the preference in base composition of the *E*. *aureum* cp genome, with an A/T abundance of 64.2%.

The SSRs within a cp genome are abundantly distributed in the non-coding regions [10, 31]. In *E*. *aureum*, those SSRs are more numerous in the non-coding regions (73.0% of all SSRs) than in the protein-coding regions (15.3% of the total; Fig 5C). Most are located in the IGS (Fig 5C), while 17 SSRs occur in protein-coding genes such as *rps19*, *rps7*, *rpoC1*, *psbI*, *ndhD*, *accD*, *ycf1*, and *ycf2* (S2 Table). The genes *ycf1* and *ycf2* contain more SSR loci than the other genes (S2 Table). Furthermore, 11.7% of the SSRs are in the introns and only 15.3% are in the protein-coding regions, accounting for 49.1% of the entire cp genome. This suggests that the SSRs are distributed unequally. The *E*. *aureum* genome has multiple repeat sequences in the *trnT*(*UGU*)-*trnL*(*UAA*) IGS and in *ycf1* (i.e., each with six repeats). This SSR information serves as a useful reference for future studies on the identification, development, and application of molecular markers for examining population genetics.

The distribution of SSRs varies among species, with genomic diversity being smaller in higher plants [56]. We also detected fewer SSRs (111) in *E*. *aureum* than in *D*. *superbus* (10,543) and *Ananas comosus* (205). However, our focus species has more than the 58 in *Colchicum autumnale* and the 56 in *Gloriosa superba*, and its SSRs are longer than 15 bp in the non-coding regions [13, 31, 40]. These characters of SSRs in *E*. *aureum* are consistent with a previous report that the majority of mononucleotide repeats is distributed universally in cp genomes when compared with other types of SSRs; that the longer SSRs are mainly located in non-coding regions; and that the number, relative abundance, and relative density of SSRs are weakly influenced by GC content in those cp genomes [56].

**Fig 6:**
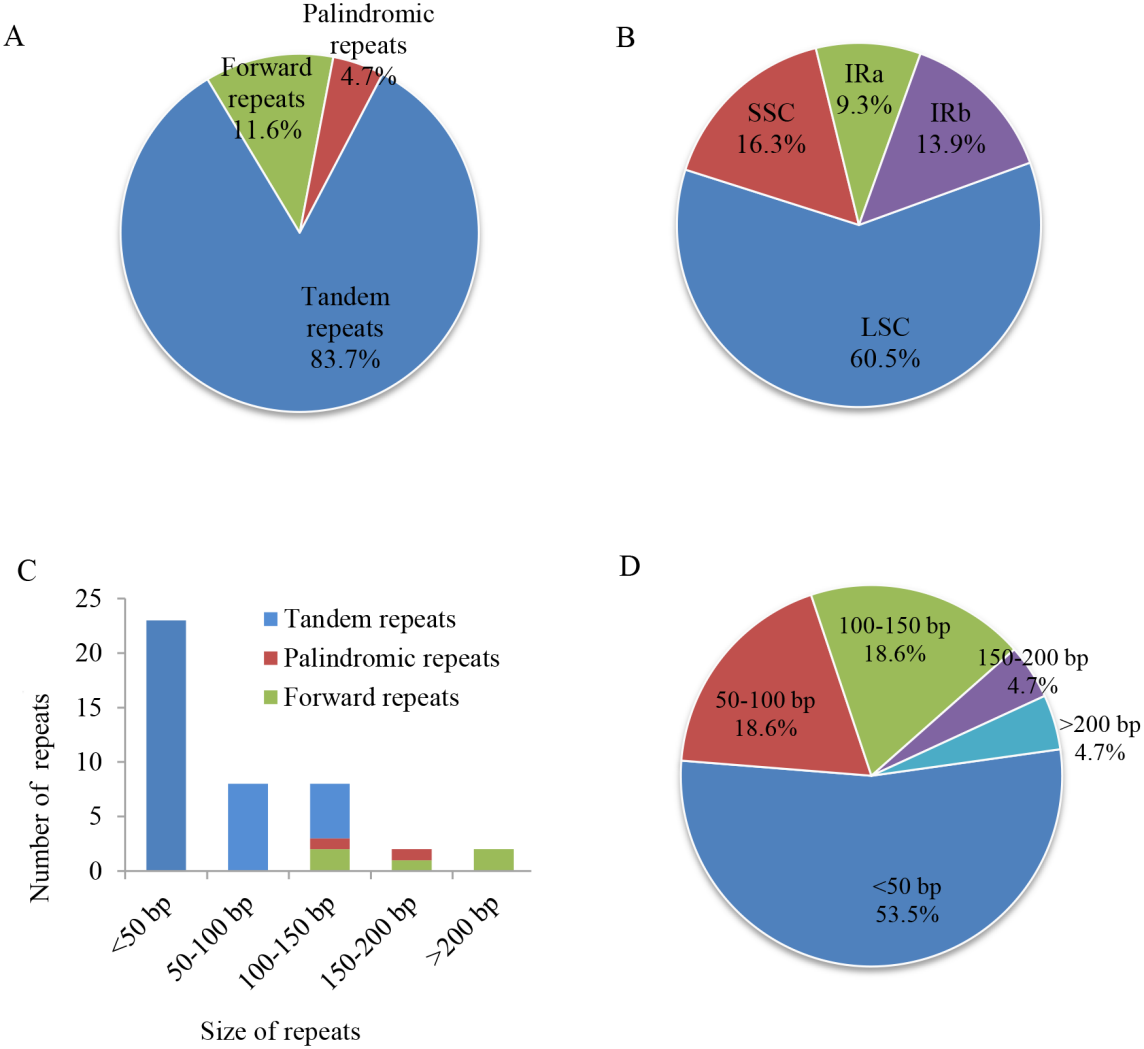
Analysis of repeated sequences in *Epipremnum aureum* chloroplast genome. (A) Sorted by type of repeat. (B) Frequencies of repeat groups. (C) Numbers of each repeat type. (D) Sorted by size of repeat.

Repeat motifs are crucial when analyzing genome recombination, rearrangement, and phylogenetic development, or when inducing substitutions and indels in a cp genome [3, 10]. The *E*. *aureum* chloroplast DNA has 43 large repeats that are longer than 9 bp (S3 Table). They include five forward repeats (11.6%), two palindromic repeats (4.7%), and 36 tandem repeats (83.7%; Fig 6A). Among them, 53.5% are shorter than 50 bp (Fig 6D). Most of the tandem repeats are less than 100 bp long while the forward and palindromic repeats are more than 100 bp in length (Fig 6C). Furthermore, 36 tandem repeats are nine to 115 bp, with most being less than 50 bp. The forward repeats are 127 to 217 bp long, while the two palindromic repeats are 137 and160 bp. Among these 43 repeats, 60.5% are located in the LSC, 16.3% in the SSC, and 23.2% in the IR regions (Fig 6B). Most (62.8%) are distributed in the IGS while 18.6% are in the protein-coding regions and 9.3% in the introns. The number, composition, and distribution of repeats in *E*. *aureum* are similar to those in *Ananas comosus* [13]. These repeat motifs have been selected for population studies and phylogenetic analysis because they are an informative source for developing markers [10, 39].

### Comparison of IR junctions among Araceae species

**Fig 7:**
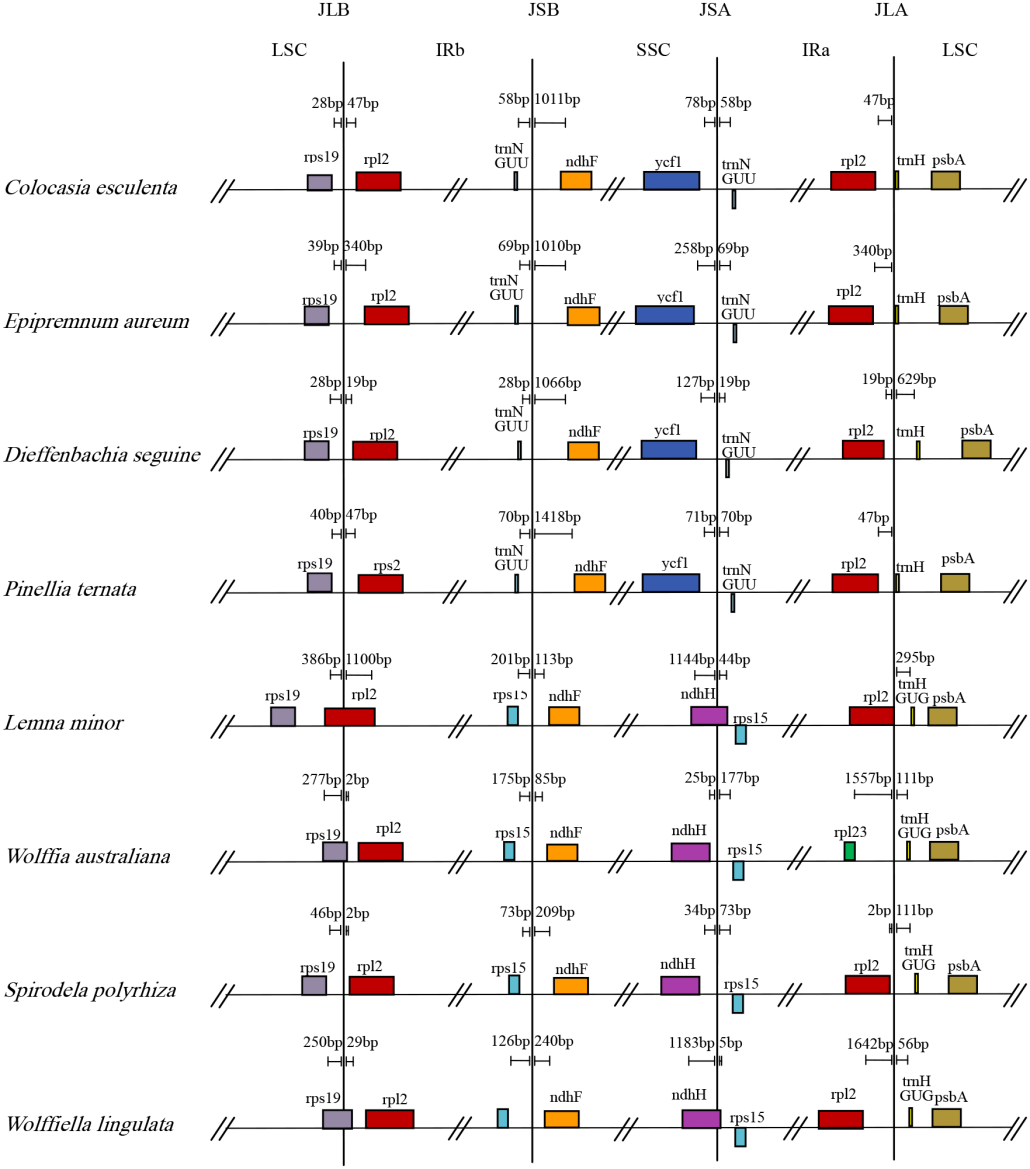
Comparison of IR boundaries among Araceae members.

Changes in genes within the IRs significantly influence the boundaries of those particular regions with the LSC or SSC region, as well as expansion and contraction of the genome [31]. We compared IR junctions among members of Araceae (Fig 7) and found that those locations are generally similar for all cp genomes. However, some notable characteristics include the following. First, the LSC/IRa boundary is within *trnH*-*rpl2*. The boundary that divides LSC/IRb fluctuates near the *rps19* to *rpl2* region with one exception, i.e., the boundary for genes from *W*. *australiana* is between *trnH* and *rpl23* due to the loss of *rpl2* in the IRa region. Second, Araceae members can be assigned to two separate groups based on their differences in SSC/IR junctions: Group 1, SSC/IRa and SSC/IRb boundaries occurring at *ycf1*-*trnN* and *ndhF*-*trnN* in *C*. *esculenta*, *E*. *aureum*, *P*. *ternata*, and *D*. *seguine*; and Group 2, SSC/IRa and SSC/IRb boundaries expand to *adhH/rps15* and *ndhF*/*rps15*, respectively, in the cp genomes of *L*. *minor*, *S*. *polyrhiza*, *W*. *australiana*, and *W*. *lingulata*. These findings are in accord with those described for members of Alismatales [22].

### Base substitution ratios among Araceae species

**Fig 8:**
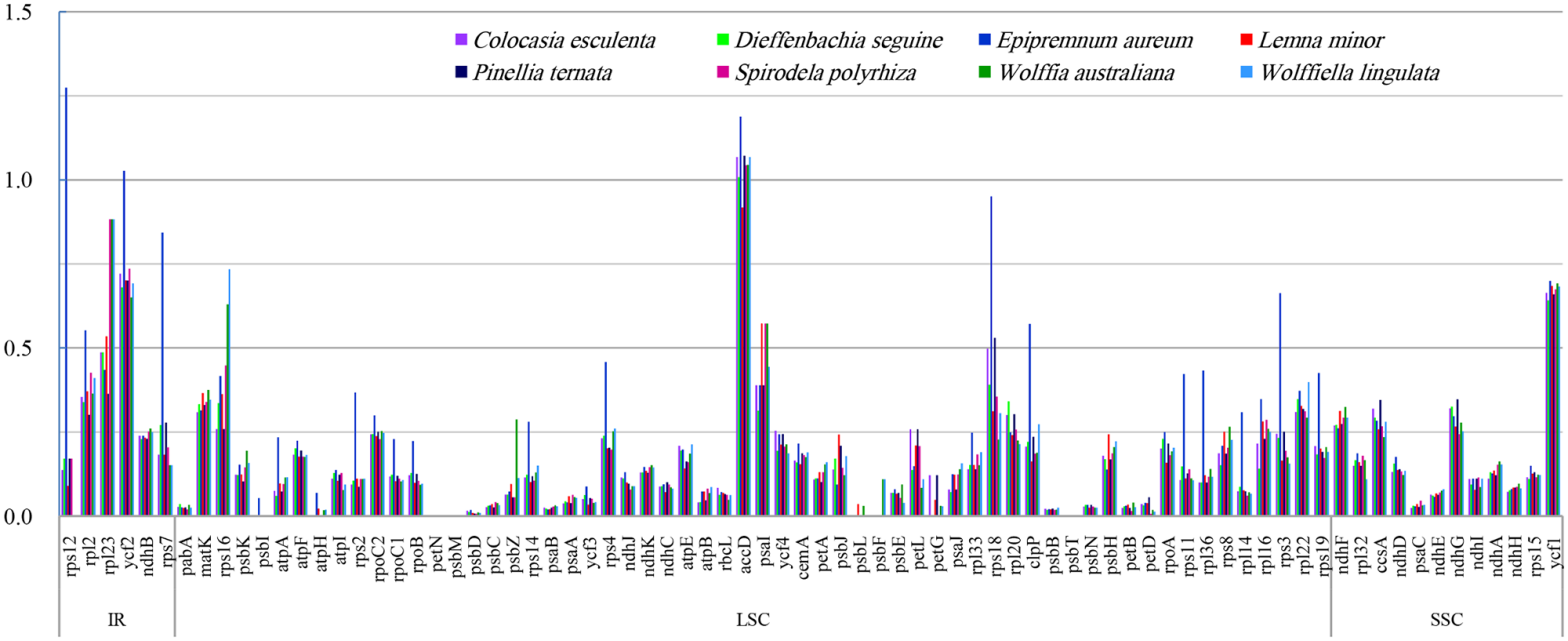
Comparison of nonsynonymous (dN) and synonymous (dS) substitution ratios among eight species of Araceae, using chloroplast genome of *Camellia crapnelliana* (Theaceae) as reference.

The nonsynonymous (dN) and synonymous (dS) substitution ratio, or dN/dS, is an important means for evaluating genome evolution [31, 57]. We analyzed dN/dS among Araceae members (Fig 8), using the genome of *C*. *crapnelliana* (Theaceae) as our reference. The majority of genes in those genomes showed ratios of less than 1.00 (the exceptions being *rpsl2*, *accD*, and *ycf2*). In the SSC region, the highest and lowest ratios were identified with *ycf1* and *psaC*, respectively, while the other ndh genes ranged from 0.10 to 0.30. Because the ratios for *rps2*, *rps3*, *rps4*, *rps7*, *rps11*, *rps12*, *rps14*, r*ps18*, r*ps19*, *rpl2*, *rpl36*, *ycf1*, *ycf2*, *clpP*, *atpA*, and *ropB* were obviously higher in *E*. *aureum* than in the other species, we examined the sequence diversity in genes that were among the exceptions. The highest ratio was calculated for *accD*, and was approximately 1.00 or larger in the LSC region, thereby indicating that this gene is not conserved in the cp genome. The ratio of 1.27 for *rps12* in *E*. *aureum* was five times higher than that in other species, and it was has two positively selected sites (including Val^39^ and Tyr^43^), showing that this gene is under positive selection in *E*. *aureum* (Table 5). In contrast, the substitution rates were low to zero in the photosynthesis-related genes *psb* (*I*, *M*, *L*, *and F)* and *petN*, which are more conserved than the other gene groups in all cp genomes because of strong functional constraints. The dN/dS ratios of *ycf2*, *ndh*, and photosynthetic genes were similar to those of colchicine plants *Colchicum autumnale* L. and *Gloriosa superba* L. [31].

**Table 5:** The selection pressure sites of *rps12* in Araceae species identified by different methods.

Methods	Purifyingly selected sites	Neutrally Evolving Sites	Positively selected sites
FEL	2(Asn^10^**, Cys^27^*)	138	0
IFEL	1(Asn^10^)*	138	1 ((3Val/3Thr)^39^)*
REL	0	139	1((1Ile/1Tyr/1Met)^43^)**
MEME	0	139	1((3Val/3Thr)^39^)**
REL(Branch-Site)			*E*. aureum**

*: p<0.05,

**: p<0.01.

Val^39^: in *D*. *seguine*, *E*. *aureum*, *P*. *ternata*; Thr^39^: in *C*. *esculenta*, *L*. *minor*, *S*. *polyrhiza*. Ile^43^: in *D*. *seguine*; Tyr^43^: in *E*. *aureum*; Met^43^: in *P*. *ternata*.

### Phylogenetic analysis

**Fig 9:**
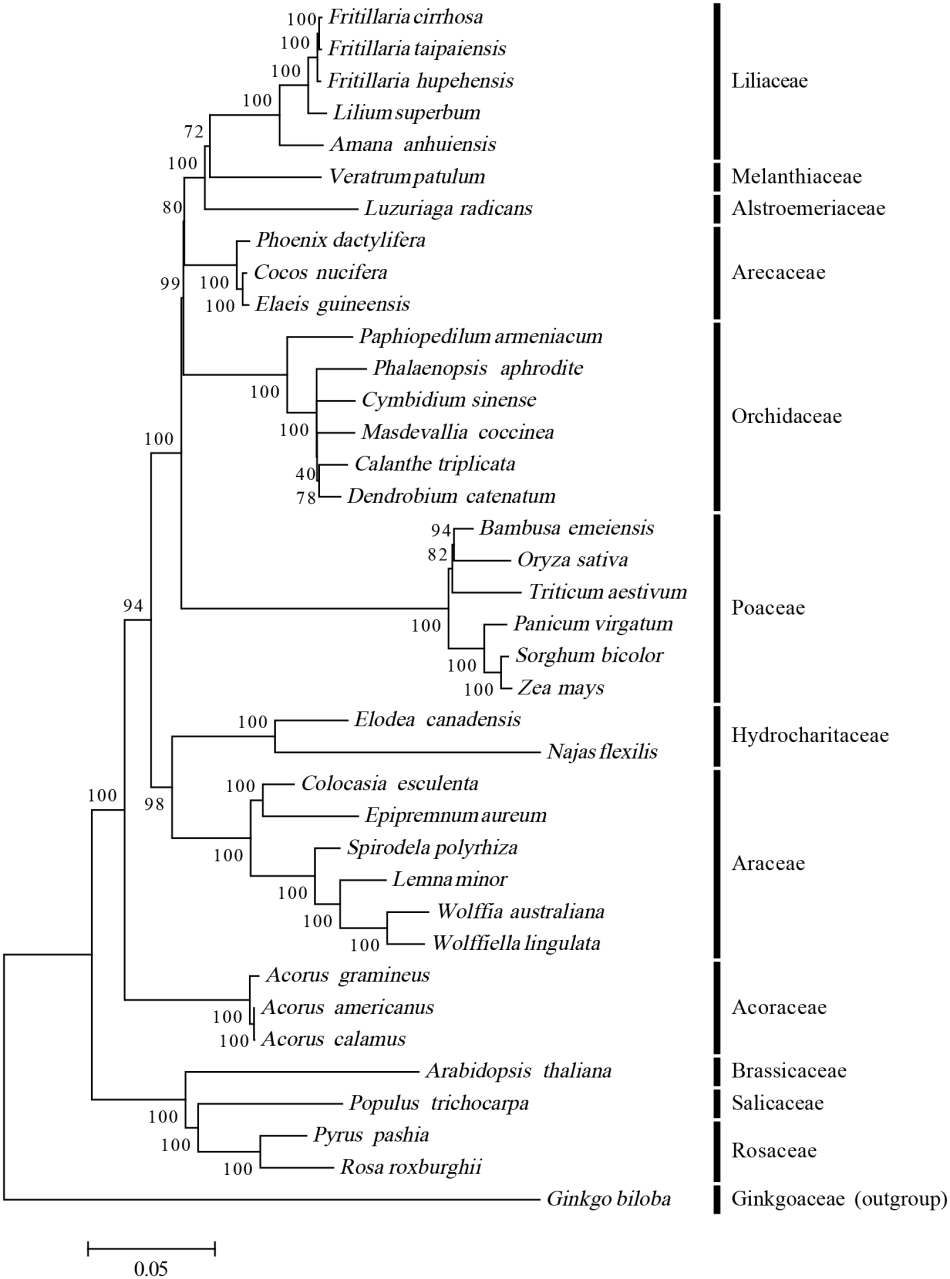
Maximum Likelihood (ML) tree based on coding sequences of 53 protein-coding genes from 38 species, using *G*. *biloba* as outgroup. Numbers on each node are bootstrap support values.

To examine the phylogenetic position of *E*. *aureum* within Araceae (Alismatales), we selected 37 species representing 13 families, i.e., Melanthiaceae, Alstroemeriaceae, Arecaceae, Orchidaceae, Acoraceae, Araceae, Hydrocharitaceae, Poaceae, Salicaceae, Rosaceae, Brassicaceae and Ginkgoaceae. *Gingko biloba* was set as the outgroup. After the cp genome sequences were downloaded from NCBI, they were aligned and a phylogenetic tree was constructed by the ML method. The position of *E*. *aureum* was situated as the sister of *C*. *esculenta*, which is a closely related species in the Araceae family (Fig 9); and *Acorus* (Acorales) was sister to Alismatales and other monocots clades. The result is consistent with the classification of *Acorus* (Acorales) according to the APG (Angiosperm Phylogeny Group) III system published in 2009 [58]. The previous report of plastid phylogenomic analyses strongly supports Acorales as sister to the remaining monocots and monophyly of Alismatales [22]. The genus *Acorus* was once placed within the family Araceae (Alismatales), though it was separated from Araceae but within Acorales, there was data that still support to place it within Alismatales [27]. Hence, the positioning of *Acorus* is still under dispute. Meanwhile, the circumscriptions of Araceae and Alismatales also have a fair amount of conflicting information [24, 26]. The controversial placement of *Acorus* and the disputed relationships within Alismatales mayhave resulted from the different data sets used [27]. With the availability of chloroplast sequences for this family, including Alismatales, future research can focus more deeply on phyletic evolution.

## Supporting information

S1 File The gene names of 53 protein-coding coding sequences used in phylogenetic tree. (DOCX)

S2 File The alignment of coding sequences of 53 protein-coding genes for phylogenetic tree. (FASTA)

S1 Table GenBank accession numbers used in phylogenetic tree. (DOCX)

S2 Table SSRs identified in the chloroplast genome of *Epipremnum aureum*. (DOCX)

S3 Table Repeats in the chloroplast genome of *Epipremnum aureum*. (DOCX)
