# Supplementary material for: The complete chloroplast genome sequence of Epipremnum aureum and its comparative analysis among eight Araceae species
Source: PLoS One. 2018 Mar 12;13(3):e0192956. doi: 10.1371/journal.pone.0192956 (PMC5846728; doi:10.1371/journal.pone.0192956)
Supplement: S1 File — (DOCX) [file pone.0192956.s001.docx]

The gene names of 53 protein-coding CDS used in phylogenetic trees:

*atpA, atpB, atpE, atpF, atpH, atpI, ccsA, cemA, matK, petA, petB, petD, petG, petN, psaA, psaB, psaC, psaJ, psbA, psbB, psbC, psbD, psbE, psbF, psbI, psbJ, psbK, psbL, psbM, psbN, psbT, rbcL, ropA, rpl16, rpl20, rpl22, rpl23, rpoA, rpoB, rpoC1, rpoC2, rps2, rps3, rps4, rps7, rps8, rps11, rps14, rps15, rps18, rps19, ycf3, ycf4.*
