# Supplementary material for: The complete chloroplast genome sequence of Epipremnum aureum and its comparative analysis among eight Araceae species
Source: PLoS One. 2018 Mar 12;13(3):e0192956. doi: 10.1371/journal.pone.0192956 (PMC5846728; doi:10.1371/journal.pone.0192956)
Supplement: S1 Table — (DOCX) [file pone.0192956.s003.docx]

GenBank accession numbers used in phylogenetic trees

| Species | Accession number | Species | Accession number |
| --- | --- | --- | --- |
| *Acorus americanus* | NC_010093.1 | *Lilium superbum* | NC_026787.1 |
| *Acorus calamus* | NC_007407.1 | *Luzuriaga radicans* | NC_025333.1 |
| *Acorus gramineus* | NC_026299.1 | *Masdevallia coccinea* | NC_026541.1 |
| *Amana anhuiensis* | NC_034706.1 | *Najas flexilis* | NC_021936.1 |
| *Arabidopsis thaliana* | NC_000932.1 | *Oryza sativa* | NC_031333.1 |
| *Bambusa emeiensis* | NC_015830.1 | *Panicum virgatum* | NC_015990.1 |
| *Calanthe triplicata* | NC_024544.1 | *Paphiopedilum armeniacum* | NC_026779.1 |
| *Cocos nucifera* | NC_022417.1 | *Phalaenopsis aphrodite* | NC_007499.1 |
| *Colocasia esculenta* | NC_016753.1 | *Phoenix dactylifera* | NC_013991.2 |
| *Cymbidium sinense* | NC_021430.1 | *Populus trichocarpa* | NC_009143.1 |
| *Dendrobium officinale* | NC_024019.1 | *Pyrus pashia* | NC_034909.1 |
| *Elaeis guineensis* | NC_017602.1 | *Rosa roxburghii* | NC_032038.1 |
| *Elodea canadensis* | NC_018541.1 | *Sorghum bicolor* | NC_008602.1 |
| *Epipremnum aureum* | NC_027954.1 | *Spirodela polyrhiza* | NC_015891.1 |
| *Fritillaria cirrhosa* | NC_024728.1 | *Triticum aestivum* | NC_002762.1 |
| *Fritillaria hupehensis* | NC_024736.1 | *Veratrum patulum* | NC_022715.2 |
| *Fritillaria taipaiensis* | NC_023247.1 | *Wolffia australiana* | NC_015899.1 |
| *Ginkgo biloba* | NC_016986.1 | *Wolffiella lingulata* | NC_015894.1 |
| *Lemna minor* | NC_010109.1 | *Zea mays* | NC_001666.2 |
