# Supplementary material for: The complete chloroplast genome sequence of Epipremnum aureum and its comparative analysis among eight Araceae species
Source: PLoS One. 2018 Mar 12;13(3):e0192956. doi: 10.1371/journal.pone.0192956 (PMC5846728; doi:10.1371/journal.pone.0192956)
Supplement: S2 Table — (DOCX) [file pone.0192956.s004.docx]

SSRs identified in the chloroplast genome of *Epipremnum aureum.*

| Number | Type | Motif | SSR | Size | Start | End | Location |
| --- | --- | --- | --- | --- | --- | --- | --- |
| 1 | Monomer | A | (A)10 | 10 | 4732 | 4741 | LSC/IGS (*trnK-UUU*, *rps16*) |
| 2 |  |  |  |  | 6967 | 6976 | LSC/IGS (*rps16*, *trnQ-UUG*) |
| 3 |  |  |  |  | 10148 | 10157 | LSC/IGS (*trnS-GCU*, *trnG-UCC*) |
| 4 |  |  |  |  | 10551 | 10560 | LSC/IGS (*trnS-GCU*, *trnG-UCC*) |
| 5 |  |  |  |  | 31659 | 31668 | LSC/IGS (*petN*, *psbM*) |
| 6 |  |  |  |  | 47775 | 47784 | LSC/IGS (*ycf*, *trnS-GGA*) |
| 7 |  |  |  |  | 51294 | 51303 | LSC/IGS (*trnT-UGU*, *trnL-UAA*) |
| 8 |  |  |  |  | 58928 | 58937 | LSC/IGS (*atpB*, *rbcL*) |
| 9 |  |  |  |  | 79624 | 79633 | LSC/IGS (*psbH*, *petB*) |
| 10 |  |  |  |  | 79686 | 79695 | LSC/IGS (*psbH*, *petB*) |
| 11 |  |  |  |  | 80671 | 80680 | LSC/IGS (*petB*, *petD*) |
| 12 |  |  |  |  | 89551 | 89560 | IRb/*rpl2** |
| 13 |  |  |  |  | 149599 | 149608 | IRa/*rps7* |
| 14 |  |  |  |  | 150301 | 150310 | IRa/IGS (*rps7*, *ndhB*) |
| 15 |  |  |  |  | 151237 | 151246 | IRa (*ndhB**) |
| 16 |  |  |  |  | 157518 | 157527 | IRa/*ycf2* |
| 17 |  |  |  |  | 157626 | 157635 | IRa/*ycf2* |
| 18 |  |  | (A)11 | 11 | 8433 | 8443 | LSC/IGS (*rps16*, *psbK*) |
| 19 |  |  |  |  | 13686 | 13696 | LSC/IGS (*atpA*, *atpF*) |
| 20 |  |  |  |  | 14891 | 14901 | LSC/*atpF** |
| 21 |  |  |  |  | 16711 | 16721 | LSC/IGS (*atpH*, *atpI*) |
| 22 |  |  |  |  | 33887 | 33897 | LSC/IGS (*trnY-GUA*, *trnE-UUC*) |
| 23 |  |  |  |  | 40451 | 40461 | LSC/IGS (*rps14*, *psaB*) |
| 24 |  |  |  |  | 50811 | 50821 | LSC/IGS (*trnT-UGU*, *trnL-UAA*) |
| 25 |  |  |  |  | 65917 | 65927 | LSC/IGS (*ycf4*, *cemA*) |
| 26 |  |  |  |  | 84949 | 84959 | LSC/IGS (*rps8*, *rpl14*) |
| 27 |  |  |  |  | 116255 | 116265 | SSC/IGS (*trnN-GUU*, *ndhF*) |
| 28 |  |  |  |  | 124332 | 124342 | SSC/IGS (*psaC*, *ndhE*) |
| 29 |  |  |  |  | 131347 | 131357 | SSC/IGS (*rps15*, *ycf1*) |
| 30 |  |  |  |  | 164566 | 164576 | IRa/IGS (*rpl2*, *trnH*) |
| 31 |  |  | (A)12 | 12 | 9234 | 9245 | LSC/*psbI* |
| 32 |  |  |  |  | 48783 | 48794 | LSC/IGS (*trnS-GGA*, *rps4*) |
| 33 |  |  |  |  | 88831 | 88842 | IRb/IGS (*rps19*, *rpl2*) |
| 34 |  |  |  |  | 130530 | 130541 | SSC/IGS (*ndhH*, *rps15*) |
| 35 |  |  | (A)13 | 13 | 29895 | 29907 | LSC/IGS (*rpoB*, *trnC-GCA*) |
| 36 |  |  |  |  | 29998 | 30010 | LSC/IGS (*rpoB*, *trnC-GCA*) |
| 37 |  |  |  |  | 31070 | 31082 | LSC/IGS (*trnC-GCA*, *petN*) |
| 38 |  |  |  |  | 51319 | 51331 | LSC/IGS (*trnT-UGU*, *trnL-UAA*) |
| 39 |  |  |  |  | 75724 | 75736 | LSC/*clpP** |
| 40 |  |  |  |  | 115797 | 115822 | SSC/IGS (*trnN-GUU*, *ndhF*) |
| 41 |  |  | (A)14 | 14 | 15931 | 15944 | LSC/IGS (*atpH*, *atpI*) |
| 42 |  |  |  |  | 32247 | 32260 | LSC/IGS (*petN*, *psbM*) |
| 43 |  |  |  |  | 48316 | 48329 | LSC/IGS *(ycf3*, *trnS-GGA)* |
| 44 |  |  | (A)15 | 15 | 47482 | 47496 | LSC/*ycf3** |
| 45 |  |  |  |  | 51824 | 51838 | LSC/*trnL** |
| 46 |  |  | (A)17 | 17 | 47515 | 47531 | LSC/*ycf3** |
| 47 |  |  | (A)24 | 24 | 10242 | 10265 | LSC/IGS (*trnS-GCU*, *trnG-UCC*) |
| 48 |  |  | (A)25 | 25 | 55479 | 55503 | LSC/IGS (*ndhC*, *trnV-UAC*) |
| 49 |  |  | (A)26 | 26 | 55514 | 55539 | LSC/IGS (*ndhC*, *trnV-UAC*) |
| 50 |  | T | (T)10 | 10 | 110 | 119 | LSC/IGS (*trnH*, *psbA*) |
| 51 |  |  |  |  | 553 | 562 | LSC/IGS (*trnH*, *psbA*) |
| 52 |  |  |  |  | 17617 | 17626 | LSC/IGS (*atpI*, *rps2*) |
| 53 |  |  |  |  | 18489 | 18498 | LSC/IGS (*rps2*, *rpoC2*) |
| 54 |  |  |  |  | 24369 | 24378 | LSC/*rpoC1* |
| 55 |  |  |  |  | 35359 | 35368 | LSC/IGS (*trnT-GGU*, *psbD*) |
| 56 |  |  |  |  | 52623 | 52632 | LSC/IGS (*trnF-GAA*, *ndhJ*) |
| 57 |  |  |  |  | 54997 | 55006 | LSC/IGS (*ndhC*, *trnV-UAC*) |
| 58 |  |  |  |  | 76632 | 76641 | LSC/IGS (*clpP*, *psbB*) |
| 59 |  |  |  |  | 95948 | 95957 | IRb/IGS (*ycf2*) |
| 60 |  |  |  |  | 96056 | 96065 | IRb/*ycf2* |
| 61 |  |  |  |  | 102337 | 102346 | IRb/*ndhB** |
| 62 |  |  |  |  | 103273 | 103282 | IRb/IGS (*ndhB*, *rps7*) |
| 63 |  |  |  |  | 103975 | 103984 | IRb/*rps7* |
| 64 |  |  |  |  | 126218 | 126227 | SSC/IGS (*ndhG*, *ndhI*) |
| 65 |  |  |  |  | 134955 | 134964 | SSC/*ycf1* |
| 66 |  |  |  |  | 134976 | 134985 | SSC/*ycf1* |
| 67 |  |  |  |  | 164023 | 164032 | IRa/*rpl2** |
| 68 |  |  | (T)11 | 11 | 5809 | 5819 | LSC/*rps16** |
| 69 |  |  |  |  | 25127 | 25137 | LSC/*rpoC1** |
| 70 |  |  |  |  | 39266 | 39276 | LSC/IGS (*psbZ*, *trnG-GCC*) |
| 71 |  |  |  |  | 49634 | 49644 | LSC/IGS (*rps4*, *trnT-UGU*) |
| 72 |  |  |  |  | 68132 | 68142 | LSC/IGS (*petA*, *psbJ*) |
| 73 |  |  |  |  | 68188 | 68198 | LSC/IGS (*petA*, *psbJ*) |
| 74 |  |  |  |  | 89007 | 89017 | IRb/IGS (*rps19*, *rpl2*) |
| 75 |  |  |  |  | 134511 | 134521 | SSC/*ycf1* |
| 76 |  |  |  |  | 134835 | 134845 | SSC/*ycf1* |
| 77 |  |  | (T)12 | 12 | 35708 | 35719 | LSC/IGS (*trnT-GGU*, *psbD*) |
| 78 |  |  |  |  | 17637 | 17648 | LSC/IGS (*atpI*, *rps2*) |
| 79 |  |  |  |  | 88672 | 88683 | LSC/*rps19* |
| 80 |  |  |  |  | 120106 | 120117 | SSC/IGS (*rpl32*, *trnL-UAG*) |
| 81 |  |  |  |  | 120160 | 120171 | SSC/IGS (*rpl32*, *trnL-UAG*) |
| 82 |  |  |  |  | 136431 | 136442 | SSC/*ycf1* |
| 83 |  |  |  |  | 164741 | 164752 | IRa/IGS (*rpl2*, *trnH*) |
| 84 |  |  | (T)13 | 13 | 61360 | 61372 | LSC/IGS (*rbcL*, *accD*) |
| 85 |  |  |  |  | 133005 | 133017 | SSC/*ycf1* |
| 86 |  |  | (T)14 | 14 | 30244 | 30257 | LSC/IGS (*rpoB*, *trnC-GCA*) |
| 87 |  |  |  |  | 59388 | 59401 | LSC/IGS (*atpB*, *rbcL*) |
| 88 |  |  |  |  | 75433 | 75446 | LSC/*clpP** |
| 89 |  |  | (T)15 | 15 | 61729 | 61743 | LSC/*accD* |
| 90 |  |  |  |  | 84926 | 84940 | LSC/IGS (*rps8*, *rpl14*) |
| 91 |  | C | (C)11 | 11 | 122903 | 122913 | SSC/*ndhD* |
| 92 |  | G | (G)12 | 12 | 79898 | 79909 | LSC/*petB** |
| 93 | Dimer | AT | (AT)6 | 12 | 76476 | 76487 | LSC/IGS (*clpP*, *psbB*) |
| 94 |  |  |  |  | 124600 | 124611 | SSC/IGS (*psaC*, *ndhE*) |
| 95 |  |  |  |  | 126395 | 126406 | SSC/IGS (*ndhG*, *ndhI*) |
| 96 |  |  |  |  | 131644 | 131655 | SSC/IGS (*rps15*, *ycf1*) |
| 97 |  |  | (AT)7 | 14 | 300 | 313 | LSC/IGS (*trnH*, *psbA*) |
| 98 |  |  |  |  | 316 | 330 | LSC/IGS (*trnH*, *psbA*) |
| 99 |  |  | (AT)8 | 16 | 33915 | 33930 | LSC/IGS (*trnY-GUA*, *trnE-UUC*) |
| 100 |  |  |  |  | 50839 | 50854 | LSC/IGS (*trnT-UGU*, *trnL-UAA*) |
| 101 |  |  | (AT)74 | 148 | 131460 | 131607 | SSC/IGS (*rps15*, *ycf1*) |
| 102 |  | TA | (TA)6 | 12 | 72756 | 72767 | LSC/IGS (*rpl33*, *rps18*) |
| 103 |  |  |  |  | 115738 | 115749 | SSC/IGS (*trnN-GUU*, *ndhF*) |
| 104 |  |  |  |  | 115752 | 115763 | SSC/IGS (*trnN-GUU*, *ndhF*) |
| 105 |  |  |  |  | 124185 | 124196 | SSC/IGS (*psaC*, *ndhE*) |
| 106 |  |  | (TA)9 | 18 | 50778 | 50786 | LSC/IGS (*trnT-UGU*, *trnL-UAA*) |
| 107 |  |  | (TA)24 | 48 | 1979 | 2026 | LSC/IGS (*psbA*, *trnK-UUU*) |
| 108 |  |  | (TA)28 | 56 | 221 | 276 | LSC/IGS (*trnH*, *psbA*) |
| 109 | Tetramer | ATAA | (ATAA)5 | 20 | 115972 | 115991 | SSC/IGS (*trnN-GUU*, *ndhF*) |
| 110 |  | TATT | (TATT)5 | 20 | 50580 | 50599 | LSC/IGS (*trnT-UGU*, *trnL-UAA*) |
| 111 |  |  |  |  | 131752 | 131771 | SSC/IGS (*rps15*, *ycf1*) |

Gene name indicates SSR that is within its protein-coding region. IGS, intergenic spacers; *, SSR within intronic region.
