# Supplementary material for: The complete chloroplast genome sequence of Epipremnum aureum and its comparative analysis among eight Araceae species
Source: PLoS One. 2018 Mar 12;13(3):e0192956. doi: 10.1371/journal.pone.0192956 (PMC5846728; doi:10.1371/journal.pone.0192956)
Supplement: S3 Table — (DOCX) [file pone.0192956.s005.docx]

Repeats in the chloroplast genome of *Epipremnum aureum*.

| Type | Number | Size  (bp) | Location | | Repeat unit |
| --- | --- | --- | --- | --- | --- |
| **F** | 1 | 217 | | LSC/IGS (*psbE*, *petL*) | GAATCAATAGCTTCTAATAATTCATGAAGGATTTTTATACTATTCATACAATAAATACAAAATCTATATCTCGTCTTTTCTTTTTTTTTCATATTGTTAAAAGTTTTTTTCTTCAATAGATCATAGGATACTTTTTTATCTCCTCATTCGAGATATTTTATTGGGCAATTACCTACTACTGAATGAGTTAAAATATAAAAATATATAAGTAAAGGAG |
|  | 2 | 212 | LSC/clpP (CDs-intron) | | CCTAGCATGAGGGAATGCTGTCCGCTCGCCCGGAGTGCCTCCGGACAGAATTAAAGATGCTATTGAAGCAGCTCTTCCTATGCATGTCGTATGTACAGCCGATTTCACATAGCTTATACTATCAAAAATAGATACTCCTCCCATTACCGATCCACCGGGGGAGTTTATAAACAAATGAAATTTCTTATGCGGATTCTCCAAATTAAGATACA |
|  | 3 | 183 | LSC/IGS *(trnE-UUC*, *trnT-GGU)* | | TTGTCAATACAATATATAATAAATATAAGTAATGTAATAACGTAATGGTATGATTAGATCCGAAAGACTTTTCCTACTTTCACGATTCTATATAATTTTTAAAAAATTTTTATTGATTTGTTCAGATGAGACATGATTCAATCAAATTTTGTAAATCTATGTTGTATTGGTACACGTATCAAT |
|  | 4 | 143 | IRb*/ycf1* | | TCATTTGGGACTTCATCGTTTGAGGTTAAGAAATGACCAACATTTGTCTCTTGATAATTTTGATATTCAAATTCTTCGGAATCATTAGGAAGTAGACTGTAAATTTTATTTATCCAGGCATCTGTAGAAGTTATTAAATCATC |
|  | 5 | 127 | IRb*/*IGS (*chlB*, *trnK-UUU*) | | ATGTATATAAATATAAAAAAATATAAATATAATTAATATATTAATATAATAAAATAAAAATATTATAATTATAAATAAATATTTAAATTTAAAATAAAATTAAAAAAAAAAAGAAATTTAAATTTTA |
| **P** | 1 | 160 | SSC/IGS (*trnN-GUU*,  *ndhF*, *rps15-ycf1*) | | ATGTATAAATTAAATTAAATAAATATGAATATAAATAAATAAATAAATAAATATAAATAAAATAATATGTAAATAATATAAAATATGATAATAATATATTAACTAAATTAAGTTATATAATATTTAAATATTAATAAATTATATTACTAATATATATATA |
|  | 2 | 137 | LSC/IGS (*ndhC-trnV-UAC*,  *atpB-rbcL*) | | GTTGTAGGTTATCCCCTTCTTTTTCTAATAGAAATACTAAAATTCTAATAGAAATACTAAAATTCTAATAGAAATACTAAAATTCTAATAGAAATACTAAAATTCTAATAGAAATACTAAAATTCTAATAGAAATAC |
| **T** | 1 | 12 | LSC/IGS (*matK*, *rps16*) | | ATATTATATGTG (×2) |
|  | 2 | 12 | LSC/IGS (*psbK*, *psbI*) | | TTTTTAATACTA (×2) |
|  | 3 | 9 | LSC/IGS (*trnG-UCC*, *trnG-UCC*) | | TAAATATTA (×3) |
|  | 4 | 9 | LSC/IGS (*trnG-UCC*,  *trnG-UCC*) | | TAATATTTA (×3) |
|  | 5 | 105 | LSC/IGS (*atpF*, *atpH*) | | ACTAAGAACTAAAAACGGGAAGGAAGAAAGCGAGAGGATCCGCTAATTACTAATCCTAAAATCCCAGAGGTATTCTCTCAATGAATAAGTAATTGTTAGAGTGAATCAGAATGGAATAGATCT (×2) |
|  | 6 | 24 | LSC/rps2 | | GATTTTTTATATAACTAGAACGAC (×3) |
|  | 7 | 39 | LSC/IGS (*rpoB*, *trnC-GCA*) | | TATATTCTGTTTACTGAATTACATAAAATTTTAGACAAC (×2) |
|  | 8 | 14 | LSC/IGS (*trnC-GCA*, *petN*) | | TTTTATTAAGATAG (×2) |
|  | 9 | 18 | LSC/IGS (*tRNA-fMet-CAU*, *rps14*) | | CCTTTCCCTTTGAAGTGC (×2) |
|  | 10 | 21 | LSC/IGS (*ycf3*, *trnS-GGA*) | | TCAAACATTAATGGATATCCT (×2) |
|  | 11 | 21 | LSC/IGS (*ycf3*, *trnS-GGA*) | | TCAAACATTAATGGATATCCT (×2) |
|  | 12 | 92 | LSC/IGS (*ycf3*, *trnS-GGA*) | | AAAAAAAAAGAAAAGATCGTCAAACATTAATGGATATCCTTCAAACATTAATGGATATCCTTAAGAAGAAAAAGAGTTTTTTATTCTTTTAC (×2) |
|  | 13 | 25 | LSC/IGS (*trnT-UGU*, *trnL-UAA*) | | TATCTAATTAATTCTAAAAAAAAAA (×2) |
|  | 14 | 20 | LSC/IGS (*ndhC*, *trnV-UAC*) | | TTCTAATAGAAATACTAAAA (×6) |
|  | 15 | 20 | LSC/IGS (*atpB*, *rbcL*) | | GTATTTCTATTAGAATTTTA (×5) |
|  | 16 | 87 | LSC/IGS (*ycf4*, *cemA*) | | AATAATATTCTTCAAGTGGTTTTTTTATTTAGCATTCATCGAAAAGAACAAATAAAGATGCAGGTCAAATAACTGAATATTTGCTTA(×2) |
|  | 17 | 57 | LSC/IGS (*petA*, *psbJ*) | | AAGATAAGATTACTATTTTTTTTTTTCGGGTCTATCTTCTTTTTTTAGATTCATAAG(×2) |
|  | 18 | 68 | LSC/IGS (*petA*, *psbJ*) | | AATAATATTTTTTTTTATTATGTTGACTAGGTAACTCGTTTTTAGGTTATGGAATGAATTAAAGCTTC(×2) |
|  | 19 | 38 | LSC/IGS (*rps18*, *rpl20*) | | AAAAATCCTTTTTTTATTGAAACATGTTCATTCATACT(×2) |
|  | 20 | 20 | LSC/IGS (*rps18*, *rpl20*) | | CACAAGATATGAGATAATCC(×2) |
|  | 21 | 118 | LSC/*petD* (intron) | | ATAGTCGATTCAAACTGAATCTTCCCTAGATACATTATTTTTAATCCATCTCAATACGGATTGTGCTAAAGATGAAGTAATTGTGATAGATTTAAAATGAAAACTTAGTCTTAGGTAG(×2) |
|  | 22 | 18 | LSC/*petD* (intron) | | ATTATATATTAAATTAGA(×2) |
|  | 23 | 26 | IRb/*rpl2* (intron) | | CGTCTTCCTCTCTTTGAAACAAGGGG(×2) |
|  | 24 | 78 | IRb/*ycf2* | | GTAGTGTTTGATCAATTACGTATTAATATTAATCAATTTTTGATTGATTACACTCAGCCACTGTCTTTCTTTTTATCC(×2) |
|  | 25 | 12 | IRb/*ycf2* | | AATCTCATAAGT0(×2) |
|  | 26 | 108 | IRb/*ycf2* | | GGATTCCTACGAGGATGAGGATTCCTTTATAGCTGTATTTGAAAATGGCAATAAGAATTTTTTTTTTAATATCATCAATCTCATAAGTAATCTCATAAGTAATCCCAT(×2) |
|  | 27 | 48 | SSC/IGS (*rpl32*, *trnL-UAG*) | | ATGATACCGAATTACGTAAACGTAATATGTAATATGTAAACATTTTAA(×2) |
|  | 28 | 91 | SSC/IGS (*ndhE*, *ndhG*) | | CTTAAGTATTTTATTAATACTAAGCTAAGTATTTAGTATTTAAGTATTTATTATATAATACTAATTATTATATTATTTTATTATTGACGAG(×2) |
|  | 29 | 9 | SSC/*rps15* | | TTTTCTTCT(×2) |
|  | 30 | 32 | SSC/IGS (*rps15*, ycf1) | | TGGATAGAAAGGTATAATATATTCCTGCACTC(×2) |
|  | 31 | 102 | SSC/*ycf1* | | TCTTATTACTGATTGTAGAATCTTTGACTTTCCCTTTTTCTTTCATTCTTCGTTCCATAACTATCATTTTTTTGTCAACCCGTCTTTCAATCTTTGAAAACC(×2) |
|  | 32 | 66 | SSC/*ycf1* | | GAGTCCGTGTTAAGGTTAAAGTTAAAGTGGAATTCATCCTTTGTTTTTTCTTTTCCATTCACTAAT(×2) |
|  | 33 | 12 | IRa/*ycf2* | | GATTACTTATGA(×2) |
|  | 34 | 108 | IRa/*ycf2* | | ATGGGATTACTTATGAGATTACTTATGAGATTGATGATATTAAAAAAAAAATTCTTATTGCCATTTTCAAATACAGCTATAAAGGAATCCTCATCCTCGTAGGAATCC(×2) |
|  | 35 | 78 | IRa/*ycf2* | | GGATAAAAAGAAAGACAGTGGCTGAGTGTAATCAATCAAAAATTGATTAATATTAATACGTAATTGATCAAACACTAC(×2) |
|  | 36 | 26 | IRa/*rpl2* | | CGCCCCTTGTTTCAAAGAGAGGAAG(×2) |

F, forward repeats; P, palindromic repeats; T, tandem repeats; IGS, intergenic spacers
